# Combining staged laparoscopic colectomy with robotic completion proctectomy and ileal pouch–anal anastomosis (IPAA) in ulcerative colitis for improved clinical and cosmetic outcomes: a single-center feasibility study and technical description

**DOI:** 10.1007/s11701-022-01466-x

**Published:** 2022-11-03

**Authors:** Dominique Lisa Birrer, Maurus Frehner, Janina Kitow, Kim-Marie Zoetzl, Andreas Rickenbacher, Luc Biedermann, Matthias Turina

**Affiliations:** 1 Department of Surgery and Transplantation, University Hospital of Zurich, Rämistrasse 100, 8091 Zurich, Switzerland; 2 Department of Gastroenterology and Hepatology, University Hospital Zurich, Rämistrasse 100, 8091 Zurich, Switzerland

**Keywords:** Ulcerative colitis, Three-stage, Procedure, Restorative proctocolectomy, Laparoscopy, Robotic surgery, Ileal pouch–anal anastomosis (IPAA)

## Abstract

Robotic proctectomy has been shown to lead to better functional outcomes compared to laparoscopic surgery in rectal cancer. However, in ulcerative colitis (UC), the potential value of robotic proctectomy has not yet been investigated, and in this indication, the operation needs to be adjusted to the total colectomy typically performed in the preceding 6 months. In this study, we describe the technique and analyze outcomes of a staged laparoscopic and robotic three-stage restorative proctocolectomy and compare the clinical outcome with the classical laparoscopic procedure. Between December 2016 and May 2021, 17 patients underwent robotic completion proctectomy (CP) with ileal pouch–anal anastomosis (IPAA) for UC. These patients were compared to 10 patients who underwent laparoscopic CP and IPAA, following laparoscopic total colectomy with end ileostomy 6 months prior by the same surgical team at our tertiary referral center. 27 patients underwent a 3-stage procedure for refractory UC (10 in the lap. group vs. 17 in the robot group). Return to normal bowel function and morbidity were comparable between the two groups. Median length of hospital stay was the same for the robotic proctectomy/IPAA group with 7 days [median; IQR (6–10)], compared to the laparoscopic stage II with 7.5 days [median; IQR (6.25–8)]. Median time to soft diet was 2 days [IQR (1–3)] vs. 3 days in the lap group [IQR 3 (3–4)]. Two patients suffered from a major complication (Clavien–Dindo ≥ 3a) in the first 90 postoperative days in the robotic group vs. one in the laparoscopic group. Perception of cosmetic results were favorable with 100% of patients reporting to be highly satisfied or satisfied in the robotic group. This report demonstrates the feasibility of a combined laparoscopic and robotic staged restorative proctocolectomy for UC, when compared with the traditional approach. Robotic pelvic dissection and a revised trocar placement in staged proctocolectomy with synergistic use of both surgical techniques with their individual advantages will likely improve overall long-term functional results, including an improved cosmetic outcome.

## Introduction

Staged restorative proctocolectomy with ileal pouch anal anastomosis (IPAA) remains the standard of care for ulcerative colitis (UC) refractory to medical therapy [1–3]. Up to date, for patients on immunosuppressive medications, a three-stage procedure is recommended to allow for safe discontinuation of immunosuppressive medications as well as reduction of inflammatory activity in the rectum and adjacent pelvic tissue upon fecal stream diversion before IPAA and thereby lower perioperative morbidity [4]. The use of minimally invasive techniques for IPAA are attractive options with an increasing body of evidence in the literature. Laparoscopic colectomy is a well-established, standardized operation with low morbidity which has safely been performed for years. Advantages of laparoscopy include unrestricted access to all four quadrants, lower postoperative pain, earlier restoration of bowel function, shorter length of stay (LOS) and lower incisional hernia rate [1, 5–7]. Similarly, completion proctectomy and IPAA can be performed safely using the laparoscopic approach [5]. Less blood loss and earlier functional recovery with lowered LOS compared to the open technique have been described (shorter hospital stay e.g.) [1, 5, 8–10]. In recent years, a growing body of literature has emerged which demonstrates distinct functional benefits of robotic over laparoscopic proctectomy in rectal cancer [11]. These include lower blood loss, shorter length of stay, and less impairment of bladder and sexual function, especially in males. However, whether robotic CP and IPAA are superior over the laparoscopic or open approach has not yet been confirmed in a prospective comparison. Since straight laparoscopy is likely associated with the same disadvantages in low pelvic surgery for UC as in rectal cancer surgery, the benefits of the robotic approach with its 3-dimensional vision, improved dexterity and range of motion are likely to translate into similar improvements in postoperative function [12].

The purpose of our retrospective study was to demonstrate the safety and feasibility of robotic CP in a three-stage procedure for UC. We furthermore aimed to analyze return to normal bowel function, length of hospital stay, and cosmetic results. Regarding the latter, special emphasis was taken on careful selection of matching laparoscopic and robotic trocar sites, to reduce the overall number of trocar incisions.

## Methods

### Patient selection

#### European Academy of Robotic Colorectal Surgery (EARCS) Database

Data of all consecutive patients who underwent robotic surgery at the University Hospital Zurich from December 2016 and August 2021 were retrieved and entered into a prospective database provided by the EARCS. Among them, 17 patients with a diagnosis of ulcerative colitis undergoing a three-stage restorative proctocolectomy were selected. Excluded were patients with synchronous colorectal cancers and patients < 18 years of age.

As a control group, ten consecutive patients undergoing same three-stage operation in a purely laparoscopic technique by the same surgeons were included in this study. All patients received regular follow-up in our outpatient clinic and at the referring gastroenterologist later. All patients provided written, informed consent. Ethical approval was waived by the local Ethics Committee of Zurich and is certified that this study was performed in accordance with the ethical standards as laid down in the 1964 Declaration of Helsinki and its later amendments Institutional review board approval was provided (BASEC Nr. 2019-00208).

### Novel combination of surgical techniques: three-stage restorative proctocolectomy with laparoscopic colectomy as stage I and robotic completion proctectomy with ileal pouch–anal anastomosis as stage II followed by closure of diverting loop ileostomy in stage III

#### Stage 1: laparoscopic total abdominal colectomy and end ileostomy (TAC + EI)

**Fig. 1:**
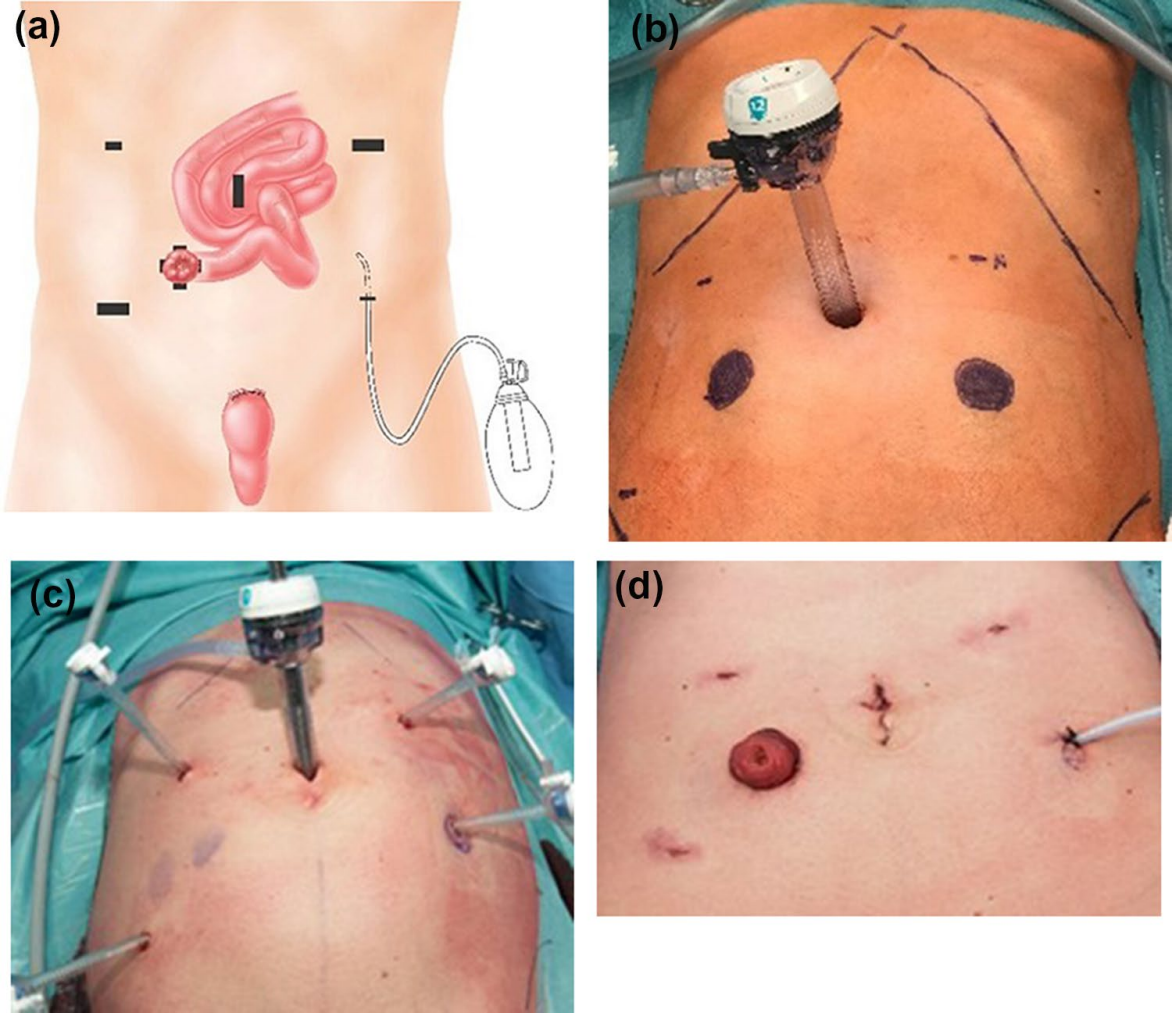
Laparoscopic total abdominal colectomy and end ileostomy (TAC + EI). **a** Four laparoscopic trocars are lined up allowing correct future placement of the intuitive da Vinci Xi system trocars. **b** Marks at the beginning of stage. **c** With trocar placement. **d** End of surgery

Laparoscopic total colectomy with terminal ileostomy was performed with a supraumbilical camera access and additional 5 mm working trocars in the left and right hemiabdomen as shown in Fig. 1a. Four laparoscopic trocars were lined up such that their future use allows for correct placement for the intuitive DaVinci Xi surgical system trocars. The colon was separated with the Endo-GIA at the level of the promontory. For this purpose, a 12 mm trocar was inserted at the future ileostomy site. The fashioning of the ostomy concluded this procedure (Fig. 1a–d).

#### Stage 2: robotic completion proctectomy, ileal pouch–anal anastomosis and diverting loop ileostomy (CP & IPAA & DLI)

The DaVinci Xi surgical system is used for the CP with IPAA with robotic ports being placed at the same sites used for the earlier total colectomy. This was achieved by alterations of the port sites used for total colectomy as shown in Fig. 1a.

**Fig. 2:**
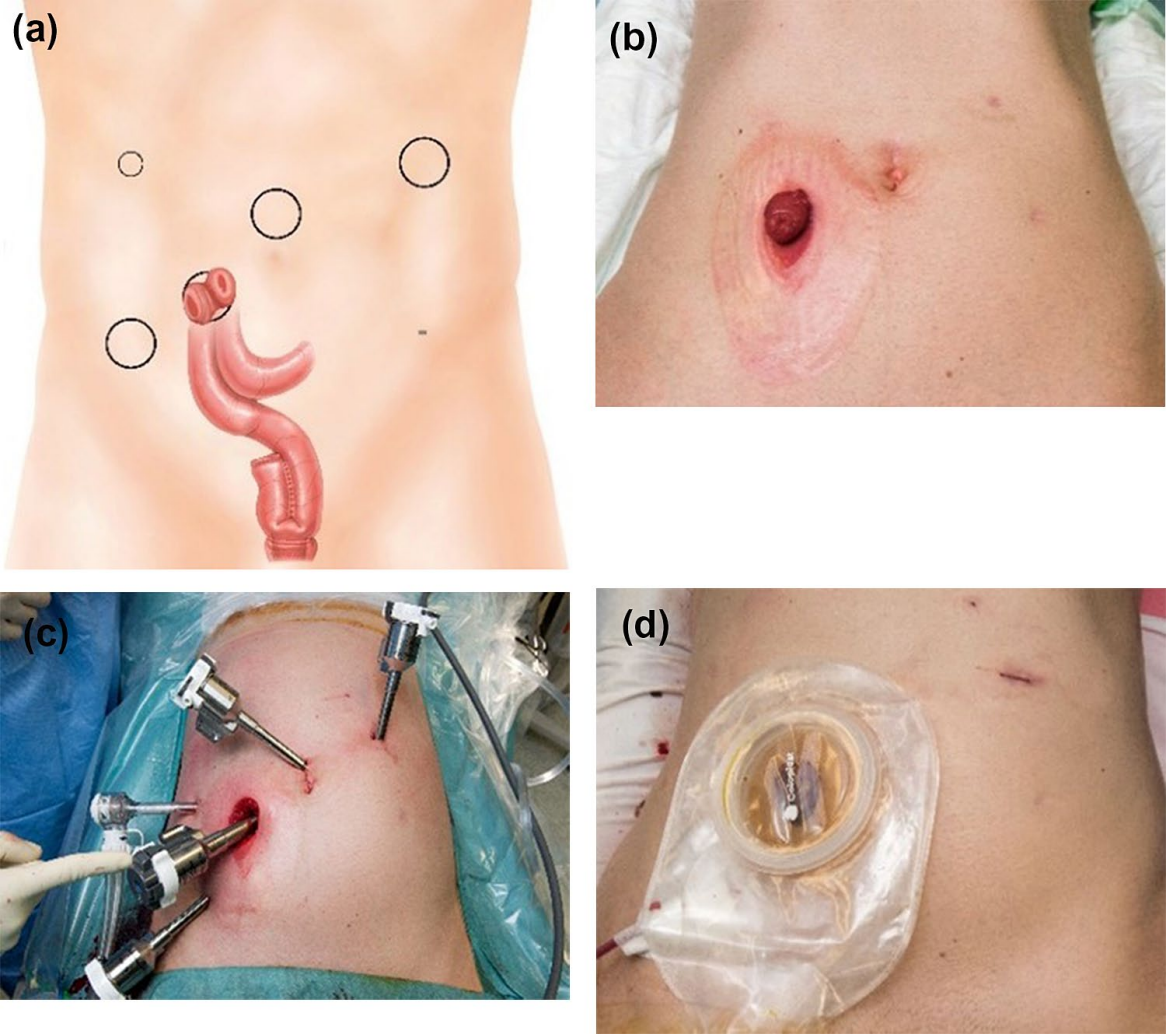
Robotic completion proctectomy, ileal pouch–anal anastomosis and diverting loop ileostomy (CP & IPAA & DLI). **a** Suggested port placement. **b** At the beginning of stage 2. **c** Trocar placement for the robotic Xi system. **d** End of surgery

Modified lithotomy position. The port sites for the robot were now determined by the existing laparoscopy scars. Docking of the robot. Identification and clipping of the superior rectal artery using robotic hem-o-loc clips. Mobilization along the posterior fascia of the mesorectum to the pelvic floor. Opening of the peritoneum laterally and anteriorly. Stepwise mobilization of the mesorectum and rectum until full mobilization to the levators has been achieved. Dissection of the distal rectum at the anorectal junction using a robotic 60 mm stapler. Retrieval of the rectum through a pfannenstiel incision of 6 cm lengths. Rotation of the robotic arms to allow access to the epigastrium. Mobilization of the mesoileum above the third part of the duodenum. Undocking of the robot. Takedown of the ileostomy. Fashioning of the j-pouch through the pfannenstiel incision using a combination of 75 mm linear staplers. Laparoscopic pouch–anal anastomosis using the da Vinci camera without docking of the robot. Identification of the terminal ileum and exteriorizing the new ileostomy. Fashioning of the ileostomy (Fig. 2a–d).

#### Stage 3: takedown of the diverting loop ileostomy

Completion ileostomy reversal was performed open, 4 months after successful IPAA.

**Fig. 3:**
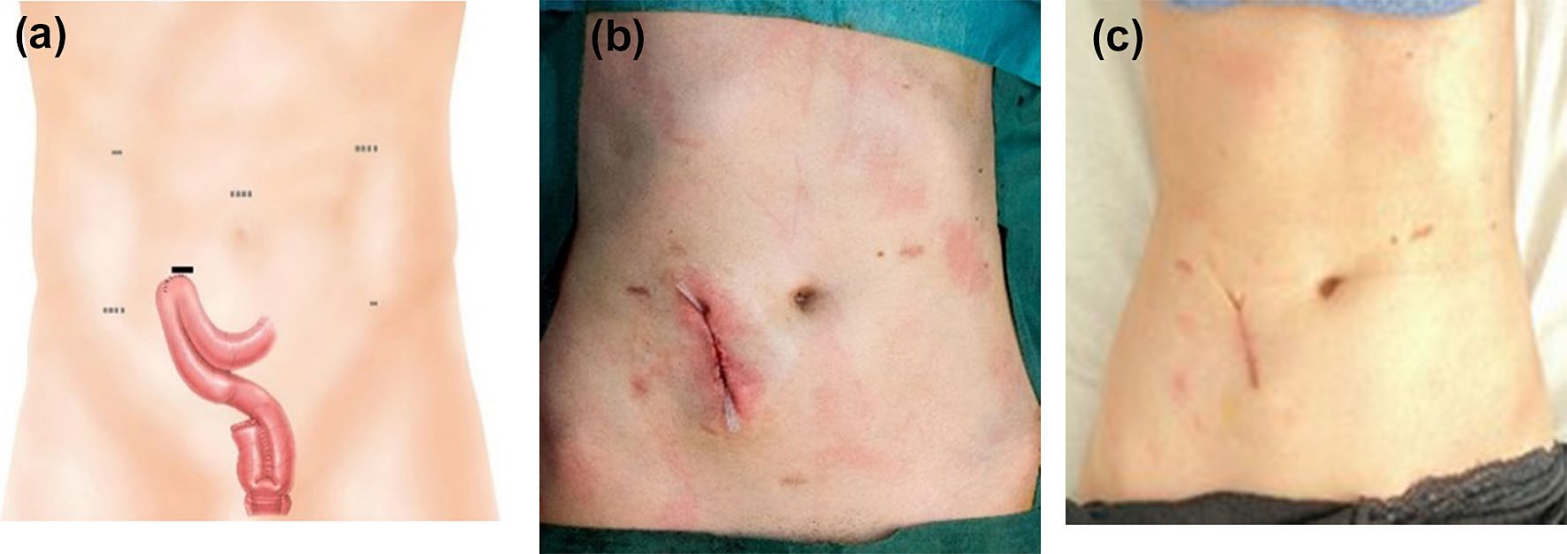
Takedown of the diverting loop ileostomy. **a** + **b** End of Surgery. **c** Four weeks after surgery

Takedown of the ileostomy was performed using either a side-to-side stapled anastomosis (75 mm linear stapler, adequate extracorporeal length of small bowel) or a hand-sewn anastomosis (short length of bowel exteriorized) (Fig. 3a–c).

### Cosmetic outcome

While we did not use an objective measurement of cosmesis, we were able to reduce the number of trocar incisions by using the same incisions for laparoscopic stage I and robotic stage II in our suggested technique.

### Statistical analysis

Since we performed a feasibility study with a technical description, no pre-specified hypotheses were made. As the parameter to estimate power of the study are not known, no power calculation was performed*.* Data are presented in a descriptive statistic manner, calculated with R [13]. Quantitative data are presented with median and interquartile range.

## Results

From January 2012 till May 2021, 17 patients were eligible and selected from the 222 robotically operated patients, for the laparoscopic group undergoing a 3-stage procedure, 10 patients were chosen.

Patients’ characteristics, procedural data, as well as morbidity for both groups are represented in Table 1. Median length of operation was not significantly different between the two groups, with 285 min [IQR 270–330] for the robotic completion proctectomy and IPAA and 270 min [IQR 243.75–299] for the laparoscopic approach. Two patients in the robotic group had to be converted to an open surgery. One conversion to open surgery occurred in a patient with inadequate reach of the small bowel to the anorectal stump and one conversion to open occurred due to intraoperative bleeding. In neither group, patients needed to stay in the intensive care unit. The return to normal bowel function and overall morbidity between the groups was comparable: the length of hospital stay (LOHS) was the same between the two groups. (Lap. 7.5 [6.25–8]; Rob. 7 [6–10]. No patient stayed longer than 1 week in the hospital, with 2 days to soft diet intake in the robotic group and 3 days in the lap. Group and no clinically relevant differences in morbidity (CCI) were observed.

**Table 1:** Demographic data

	Overall	Laparoscopic IPAA	Robotic IPAA
Demographic variables			
*n*	27	10	17
Age [median (IQR)]	36 [24.50–42]	28.50 [23.25–37]	38 [31–46]
Gender = female (%)	9 (33.3)	3 (30)	6 (35.3)
BMI (median [IQR])	23.40 [22–26.60]	23.05 [22.18–25.75]	24.10 [22–26.80]
ASA (%)			
I	4 (14.8)	4 (40.0)	0 (0.0)
II	21 (77.8)	6 (60.0)	15 (88.2)
III	2 (7.4)	0 (0.0)	2 (11.8)
In-hospital data			
Duration of surgery [median (IQR)]	280 [263.50–321.50]	270 [243.75–299]	285 [270–330]
Reason intraoperative event (%)			
Malfunctioning of robot	1 (3.7)	0 (0.0)	1 (5.9)
Conversion = yes (%)	2 (7.4)	0 (0.0)	2 (11.8)
Days to soft diet [median (IQR)]	3 [2–4]	3 [3–4]	2 [1–3]
Pathology report (%)			
UC	24 (88.9)	9 (90.0)	15 (88.2)
UC and adenocarcinoma	2 (7.4)	1 (10.0)	1 (5.9)
UC and neuroendocrine carcinoma	1 (3.7)	0 (0.0)	1 (5.9)
Length of hospital stay [median (IQR)]	7 [6–10]	7.50 [6.25–8]	7 [6–10]
Re-operation during hospital stay (bleeding)	1 (3.7)	0 (0)	1 (5.9)
Post discharge			
Major complications (30 po days, Clavien–Dindo class)			
0	24 (88.9)	9 (90.0)	15 (88.2)
3a	2 (7.4)	1 (10)	1 (5.9)
3b	1 (3.7)	0 (0)	1 (5.9)
CCI [median (IQR)] 90 pod	8.70 [8.70–22.6]	21.75 [2.17–22.6]	8.70 [8.70–27.60]
Type and treatment of complications 30 pod (%)			
Pleural and pericardial effusion (polyserositis)	1 (3.7)	0 (0)	1 (5.9)
Bleeding from rectum; endoscopically clipped	1 (3.7)	0 (0)	1 (5.9)
Abscess; CT-controlled drainage	1 (3.7)	1 (10.0)	0 (0)
Hospitalization, conservative ileus treatment	1 (3.7)	1 (10.0)	0 (0)
Gastric tube/antibiotic treatment	1 (3.7)	0 (0)	1 (5.9)
Pelvic bleeding; reoperation	1 (3.7)	0 (0)	1 (5.9)
Mortality = 1 (%)	0(0)	0 (0)	0 (0)
Re-admission within 90 po days = 1 (%)	6 (22.2)	2 (20.0)	4 (23.5)

*UC* ulcerative colitis, *CA* carcinoma, *CCI* comprehensive complication index, *po* postoperative

### Postoperative complications

Within 90 days, in both groups, there was one anastomotic leakage treated via percutaneous drainage and one pouchitis was treated with oral antibiotics. One patient in the robotic group had to be readmitted and was treated non-operatively for mechanical small bowel obstruction. Another patient had a small bleeding which was diagnosed and treated endoscopically (IIIa).

### Cosmetic results

Using the suggested trocar placement, with fewer incisions, the number of trocar scars could be kept to a minimum of 6 (Figs. 1, 2, 3). Patient perception of the cosmetic results was favorable in all patients.

## Discussion

The purpose of this analysis was to demonstrate the safety and feasibility of a modified combined laparoscopic and robotic staged restorative proctocolectomy. Return to normal bowel function, length of hospital stay, as well as morbidity, are comparable between the classical three-stage procedure with laparoscopy only for stage I and II and our modified combined technique.

Using laparoscopy for the colectomy and the robotic system for the rectum through the same trocar incisions allows for combining the advantages of laparoscopy for the total colectomy with the robotic dissection in the low pelvis without need for additional incisions. This also leads to an objective reduction in scars, which means an improved cosmetic result for the patients. Given the limitations of today’s operating robots with respect to its limited maneuverability in all four quadrants, laparoscopy is at an advantage for the first stage. Despite several reports of successful total colectomy using the da Vinci system, the downside of this technique includes impaired maneuverability and repetitive realignment of robotic arms, typically causing longer operating times and is, therefore, considered to be disadvantageous for this operation by the authors [14]. In contrast, it is the pelvic dissection during the completion proctectomy where the robotic system can reveal its genuine benefits, such as improved dexterity, better visualization and higher precision of the surgical dissection.

In our report, we suggest a trocar layout which allows laparoscopic total abdominal colectomy and end ileostomy (TAC + EI) and robotic completion proctectomy, ileal pouch–anal anastomosis and diverting loop ileostomy (CP&IPAA&DLI) using the same incisions. Therefore, this trocar layout may lead to increased satisfaction, as many non-cancer UC patients are young females, where cosmesis is a specifically relevant aspect. Our conversion rate of 11.8% is comparable to robotic procedures and in line with several reports where 5–12% of procedures were converted to open [15–20].

Several reports have shown superior functional outcomes following robotic proctectomy for cancer compared to the laparoscopic technique [21, 22]. Potential benefits of using a robot for colon and rectal cancer was analyzed in a network meta-analysis including over 40 studies [17]. The authors looked at blood loss, ileus, mortality lowest and shortest length of hospital stay in robotic group compared to open. No difference was seen in anastomotic leak rates. The authors drew the conclusion that robot-assisted colorectal surgery might me a better treatment for patients with CRC, due to several benefits, such as reduced intraoperative blood loss, shorter hospital stay and early recovery [9, 17, 18, 23].

A clear difference in urinary and sexual function could be seen in a large prospective study by Kim et al. where quality of life and functional outcome were assessed over 4 years, including 260 patients. Quality of life was comparable in both groups but the robotic approach for TME was associated with lower IPSS scores (improved bladder function) at 6 and 12 months, whereas no difference was seen in female patients. Erectile dysfunction in males showed recovery to normal within 6 months in the robotic group compared to 12 months in the lap. group [21]. On the other hand, the ROLARR randomized controlled trial, comparing the risk for conversion to open laparotomy between robot-assisted and laparoscopic surgery for rectal cancer, could not show a statistically significant difference between the groups (overall 10.1% conversion rate). Further analysis performed for secondary endpoints including postoperative complications, 30-day mortality, bladder and sexual function, could not demonstrate a superior outcome compared to the laparoscopic control group [19].

Albeit some studies demonstrated improved functional outcome for surgery of the rectum, they come with the drawback of higher costs and longer operation times and a high heterogeneity of study designs [24, 25].

Although evidence is lacking for the superiority of robotic proctectomy in UC, the strength of our study is to demonstrate that robotic completion proctectomy may well be combined with a prior laparoscopic total colectomy, providing that dedicated placement of trocars is ensured, with suitable positioning for both laparoscopic colectomy and robotic proctectomy. Aside from high patient satisfaction, our results show comparable complication rates and return to normal bowel function, in line with established techniques. There might be an additional long-term functional benefit (bladder/sexual function), which may be of even greater importance for young UC patients with a higher functional expectation and longer life expectancy than the typically older rectal cancer patients. The limits of this non-randomized and retrospective study are the relatively small number of patients in our single-center setting.

## Conclusion

With the increasing number of trained robotic colorectal surgeons, more UC patients will have an opportunity to benefit from this technique. Our current report provides a rationale by which to combine straight laparoscopy and robotic surgery in staged restorative proctocolectomy for UC. Using this suggested approach, both techniques can be used to their individual technical advantages and may potentially produce better functional and cosmetic results compared to previous techniques.

## Data Availability

Due to the sensitive nature of the data in this study, raw data would remain confidential and would not be shared.

## Abbreviations

CA: Carcinoma
CCI: Comprehensive complication index
CP: Completion proctectomy
UC: Ulcerative colitis
DLI: Diverting loop ileostomy
EARCS: European Academy of Robotic Colorectal Surgery
EI: End ileostomy
IPAA: Ileal pouch–anal anastomosis
LOS: Length of stay
Pod: Postoperative day(s)
TAC: Total abdominal colectomy
